# An exploratory study of psychological and decision-making outcomes among family members of critically ill patients

**DOI:** 10.1371/journal.pone.0334554

**Published:** 2025-11-05

**Authors:** Marym M. Alaamri, Shoroug Shaker Darweesh, Sarah Wasl Algabasani, Sarah Mohammad Alsubiei, Nora Jaber Alsolami, Hanan Mohsen Alhaddad, Afnan Tunsi, Aisha Alhofaian

**Affiliations:** Department Medical Surgical Nursing, Faculty of Nursing, King Abdul-Aziz University, Jeddah, Saudi Arabia; Public Library of Science, UNITED KINGDOM OF GREAT BRITAIN AND NORTHERN IRELAND

## Abstract

**Introduction:**

Family members of critically ill patients are often required to make complex medical decisions under emotional distress, which may affect their decision-making capacity. It is well known that psychological distress can interfere with cognitive processes that are essential for decision-making; indeed, depression and anxiety constitute some examples of such distress.

**Aim:**

This exploratory study examined psychological outcomes among family members of critically ill patients, specifically depression, anxiety, and stress. It also explored the relationships between these outcomes and decision-making self-efficacy.

**Methods:**

The study included 90 family members of intensive care unit patients. Demographic data was collected, and participants completed the Depression, Anxiety, and Stress Scale (DASS-21) and a measure of decision-making self-efficacy. Correlation analysis was used to analyze the data.

**Findings:**

The results showed participants suffering from varying levels of psychological distress, ranging from moderate to very high levels. To be specific, 61.1% of the respondents reported experiencing moderate to severe depression; furthermore, 67.2% of them suffered from moderate to severe levels of anxiety while 58.3% experienced moderate to severe stress. Statistical analysis revealed there was a significant inverse correlation between levels of distress and decision-making self-efficacy (r = −0.42, p < 0.001).

**Conclusion:**

Psychological distress may affect the confidence of family members in medical decision-making. The findings emphasize the need for psychological support, with structured opportunities to aid in the decision-making capacity in critical care settings.

## Introduction

With a population of nearly 30 million and an annual growth rate of 2.7%, Saudi Arabia experiences a high demand for intensive care services, with approximately 2 million ICU admissions annually [1]. Although the field of critical care involves primarily patient care,the psychological impact on family members can be neglected [2]. When critically ill patients are unable to participate in treatment decisions, family members can be thrust into situations where they are required to make complex, emotionally laden medical decisions on behalf of patients [3]. These situations can leave family members experiencing high levels of emotional stress and, consequently, psychological distress [4].

### Psychological distress and medical decision-making

Psychological distress is a multidimensional construct that encompasses symptoms of stress, anxiety, and depression. Each one of these can disrupt, in its own manner, the cognitive and emotional mechanisms that support effective decision-making [5,6].

According to research by Pochard et al. (2005) and Bolosi et al. (2018), depression which is persistent sadness along with loss of interest, is related to lack of motivation, negative thinking, and irresolution which in turn is likely to diminish self-efficacy during medical decision-making. Anxiety is defined as excessive worry and increased physiological activation, which, as Yeun (2021) and Johnson et al. (2019) point out, can distort decision-making processes through increased risk aversion, overestimation of potential negative results, and diminished confidence in one’s decisions [7].

Stress, which is a feeling of pressure, occurs when demands and resources to deal with them are out of balance. In emergency rooms (ERs), high levels of stress have been associated with the inability to focus, a decrease in short-term memory, and the inability to evaluate risks, all of which are essential in maintaining lucidity when making ER decisions [8]

When combined, these factors pose significant obstacles to family members’ ability to make decisions when serving as surrogate decision-makers in the intensive care unit. Their worldwide significance is highlighted by the fact that previous research conducted in both Western and Middle Eastern contexts has regularly documented high levels of these symptoms among family members of intensive care unit patients. In a Riyadh study, family members reported anxiety in 76.5% of cases, depressive symptoms in 72.8%, and stress in 61.5% [9]^.^ Similarly, first-degree relatives of patients with brain death in ICUs showed high levels of depression (75%), anxiety (76.8%), and stress (70.1%) in Iran [10]. These numbers bear testimony for the urgent need for psychological support and interventions in faced-or-families.

In another study, the participants were family members of patients at high risk of death in the ICU, where 57% experienced moderate to severe traumatic stress, 70% depressive symptoms, and 80% anxiety [10]. These findings illustrate an unforgettable burden on these families emotionally.

A systematic review done in 2019 assessed the rates of depression, anxiety, and post-traumatic stress disorder (PTSD) among family members of ICU patients [11]. The prevalence rates ranged from 2% to 80% for anxiety, 4% to 94% for depression, and 3% to 62% for PTSD. Featured with these wider ranges is the complexity of psychological outcomes among diverse populations and the consequent necessity for context-specific research and suitable interventions

Furthermore, previous research had noted that psychological distress, mainly anxiety and depression, would impair a family member’s confidence and increase his or her decisional conflict [12]. Xing et al., 2024, concluded that even if decision aids raise knowledge, high levels of distress remain and hinder their effect on decisional confidence [6]. Hence, the present study focuses on the probable negative relation between psychological distress and decision-making self-efficacy in family members of ICU patients.

This study aims to explore the relationship between psychological outcomes and decision-making self-efficacy among family members of critically ill patients. By investigating this relationship, we hope to contribute to a better understanding of the experiences and needs of family members to improve their well-being and ability to make informed decisions during these challenging times. The objectives of this study were to (1) assess the prevalence of depression, anxiety, and stress among family members of ICU patients, and (2) examine whether these psychological outcomes are associated with decision-making self-efficacy. Referring to previous studies showing low decision-making competence concomitant with psychological distress), the following hypothesis has been proposed to the second goal:

H1. Higher levels of depression, anxiety, and stress will be associated with lower decision-making self-efficacy.

## Methods

### Design and settings

A quantitative, descriptive, cross-sectional design was proposed for this study. The study protocol was approved by an institutional review board before the distribution of the survey. Because of restrictions and physical distancing measures imposed during the COVID-19 pandemic, data were collected from family members of critically ill patients through an online survey.

### Participants and sample size

A convenience sample of 90 adult family members of critically ill patients was recruited. Inclusion criteria required participants to be 18 years or older, have a family member admitted to the ICU for ≥48 hours, and have visited the patient at least once. Pediatric caregivers were excluded from this study due to evidence suggesting that caregiving dynamics and psychological responses differ significantly when the patient is a child. For instance, the emotional experiences of parents caring for critically ill children—particularly those with complex conditions are shaped by unique challenges, including long-term caregiving roles, developmental concerns, and intense emotional attachment [13].

The rationale for using a convenience sample was to ensure accessibility to participants during the study period while adhering to the inclusion requirements. To ensure adequate power, the following statistical parameters were used: a medium effect size of 0.30, an alpha level of 0.05, and a power of 0.80. A total of 85 subjects was required to address research questions [14].

### Data collection

The online survey was created using Google Forms. The survey was conducted within the period of March 16, 2021, to April 22, 2021.The survey comprised six sections: the first three sections were about the inclusion criteria and eligibility to take part in the research, the fourth section contained demographic data, the fifth section included the DASS-21 scale, and the last section consisted of the FDMSE scale. Participants were presented with a digital informed consent document describing the purpose of the study, procedures, possible risks, potential benefits, confidentiality protections, and right to withdraw from the study. Participants could only proceed to the questionnaire after providing electronic consent. The questionnaire was distributed through social media platforms—WhatsApp, Instagram, and Twitter—on the internet. The survey was designed based on the inclusion criteria, so if the participants met the inclusion criteria, they could proceed to complete the survey; if not, the attempt was automatically closed. Data collection occurred over a 5 week period, with each survey requiring approximately 20 minutes to complete.

### Ethical considerations

The approval for conducting this study has been obtained from the Research Ethics Committee at the Faculty of Nursing, King Abdulaziz University (KAU) (Reference No: 2B.90) following the guidelines defined in the Declaration of Helsinki. All participants were contacted electronically and obtained informed written consent. Informed consent was obtained electronically prior to accessing the survey. The consent form provided clear information outlining the study’s purpose, inclusion as well as exclusion criteria, and highlighted that participation was voluntary, thus allowing participants the option to agree or decline. No direct advantages or reimbursement were offered by participation in this study. The study has minimal risks, but one or two questions may remind participants about the critical condition of their loved ones, which may lead to psychological distress. Furthermore, participants were assured that their responses in the online survey would be kept anonymous and confidential.

### Instruments and measures

#### Demographic data.

Sociodemographic information was gathered to describe the sample in detail and investigate descriptive patterns in psychological outcomes and self-efficacy in making decisions. Sociodemographic information included the following: age, gender (male, female), marital status (single, married, or divorced), relation to the patient (spouse, sibling, child, or other), socioeconomic level (low, moderate, or high), educational level (uneducated, elementary, high school, or bachelor’s degree), and length of stay (> 7 days or ≤ 7 days) in a critical care unit. The level of patient consciousness was viewed as a contextual factor as it may have implications for family members’ psychological responses. In this research, “semiconscious” refers to a clinical state where the patient demonstrates limited responsiveness to external stimulation but is not wholly unconscious. Family members of patients in states of diminished consciousness often report increased distress because of perceived vagueness, lack of communication, and uncertainty regarding the patient’s prognosis [8].

#### Psychological outcomes.

The psychological outcomes were examined using the Depression, Anxiety, and Stress Scale (DASS-21). The scale consists of three subscales. Each subscale consists of seven items. DASS-21 uses four rating scores from 0 (*did not apply to me at all*) to 3 (*applied to me very much or most of the time*). The score is calculated by summing all the items on each subscale. The maximum score is 42, yet the lower scores are better. In this study, the Arabic version of DASS-21 was used. It has been widely used in clinical and caregiving populations, with strong psychometric properties in Arabic-speaking contexts [15]. The Arabic version has established a high level of internal reliability consistency with a Cronbach’s alpha coefficient of 0.93 for depression, 0.90 for anxiety, and 0.93 for stress. Additionally, the overall internal reliability consistency of the Arabic version was.86 [15]. In the current study, the DASS-21 Arabic version had a Cronbach’s alpha of 0.95. The Depression, Anxiety and Stress Scale (DASS-21) has solid psychometric properties establishing its sensitivity for emotional distress in a clinical and caregiving sample. DASS-21 also utilizes validated subscales, allowing for three related constructs to be assessed simultaneously. The Family Decision-Making Self-Efficacy (FDMSE), created for the specific purpose of assessing confidence in end-of-life decision-making contexts, is both reliable and valid for ICU and end-of-life care contexts [16].

#### Family Decision-Making Self-Efficacy (FDMSE) scale.

This scale consists of 13 items that are evaluated by a 5-point Likert scale from 1 (*cannot do at all*) to 5 (*certain I can do*), with higher scores referring to greater decision-making self-efficacy. This scale has established validity and reliability. The content validity was established by an expert panel experienced with end-of-life decision-making, including a doctoral prepared nurse, a psychiatrist, and an internal medicine specialist. The internal consistency of the FDMSE was high at Cronbach’s alpha = .95 [16]. The scale was translated into Arabic for this study. The forward translation was performed through the Certified Translation Center in Jeddah. Then, backward translation from Arabic back to English was performed by two independent bilingual translators without access to the original English. The back-translated version was compared with the original by two bilingual assistant professors from the expert panel and the first author. No errors were identified after comparing both versions. In the current study, the FDMSE Arabic version had a Cronbach’s alpha of 0.95. This scale was specifically developed in an end-of-life and ICU context, where family members must make treatment decisions under stress full conditions [16]. It directly assesses confidence in individual’s own abilities to make and advocate for decisions, so it fits theoretically with the term of reference of this study.

### Statistical analyses

The statistical package SPSS version 25.0 (IBM, Armonk, NY) was used to run the statistical analyses for this study. Descriptive statistics conducted for demographics and study variables included measurements of central tendency (mean), variability (standard deviation), and shape of distribution [17]. The Pearson correlation coefficient (*r*) was used to answer the following research question: What is the relationship between psychological effects (depression, anxiety, and stress) and decision-making self-efficacy?

## Results

### Sample characteristics

In the current study, a total of 90 family members were enrolled. The description of the sociodemographic data is presented in Table 1. The sample consisted of adult family members older than 18 years who had a family member admitted to the ICU; 47% of the participants had their family member still at the ICU and more than half had been discharged for a week or less. Most of the sample (70%) were female and 49% were single. Additionally, 59% of family members held bachelor’s degree, 23% held high school diplomas, 11% held postgraduate degrees, and 4% were not educated. Most of the family members enrolled in this study were siblings (36%) or children (27%). Regarding the length of a patient’s stay in the ICU, 26.7% stayed for 1–7 days and 28.9% for almost a month. More than half (64%) visited their family member more than three times. Furthermore, nearly half of the patients were semiconscious (44%).

**Table 1:** Sociodemographic data (n = 90).

Variables		No.	%
**Have you ever cared for a patient in the ICU?**	Yes	90	100.0
	No	0	0.0
**Age**	Older than 18 years old	90	100.0
	Younger than 18 years old	0	0.0
**Is the patient currently staying in the ICU?**	No, discharged for a week or less	47	52
	Yes, currently hospitalized	43	47.8
**Gender**	Female	63	70
	Male	27	30
**Marital status**	Single	44	48.9
	Married	38	42.2
	Widower	2	2.2
	Divorced	6	6.7
**Educational level**	Uneducated	4	4.4
	Middle school	2	2.2
	High school	21	23
	Bachelor’s degree	53	58.9
	Postgraduate degree	10	11.1
**Socioeconomic status**	High	21	23.3
	Medium	65	72.2
	Low	4	4.4
**Length of patient stay in the ICU**	1-7 days	34	37.7
	8–14 days	10	11
	15–21 days	8	8.8
	22–30 days	27	30
	31–37 days	5	5.5
	2 months	1	1.1
	3 months	3	3.3
	6 months	2	2.2
**How many times have you visited the patient in the ICU?**	One visit	12	13.3
	Two visits	8	8.9
	Three visits	7	7.8
	More than three visits	51	56.7
	Daily	5	5.6
**Patient’s level of consciousness**	Conscious	33	36.7
	Semiconscious	40	44.4
	Unconscious	17	18.9

### Level of depression, anxiety, and stress among family members of patients with critical illnesses

In total, the study included 90 family members. Out of the 68 participants displaying depression symptoms, 11.1% t reported mild, 23.3 percent moderate, 18.9% severe, and 22.2% extremely severe depression, as shown in Table 2. The prevalence of anxiety was 72.2%. Of the 65 family members with a symptom of anxiety, 1.1% reported mild anxiety, 13.3% reported moderate anxiety, 11.1% reported severe anxiety, and 46.7% reported extremely severe anxiety. The prevalence of stress was 66.7%. Of the 60 family members with a symptom of stress, 3.3% reported mild stress, 32.2% reported moderate stress, 15.6% reported severe stress, and 15.6% reported extremely severe stress.

**Table 2:** DASS21.

	Depression		Anxiety		Stress	
	N	%	N	%	N	%
Normal	22	24.4	25	27.8	30	33.3
Mild	10	11.1	1	1.1	3	3.3
Moderate	21	23.3	12	13.3	29	32.2
Severe	17	18.9	10	11.1	14	15.6
Extremely severe	20	22.2	42	46.7	14	15.6

### Examination of the relationship between psychological outcomes and decision-making self-efficacy

Pearson’s correlation coefficient was used for the analysis, and the results revealed that there were no significant relationships between decision-making self-efficacy and the DASS-21 scale and subscales, as shown in Table 3. The *p* value for all correlations was greater than 0.05, indicating that the relationships observed were not statistically significant. This suggests that decision-making self-efficacy among family members of critically ill patients is not significantly related to their levels of depression, anxiety, and stress. Although this finding may seem surprising, it highlights the complexity of the psychological outcomes experienced by family members in such challenging situations. Pearson’s correlation was chosen as an initial exploratory analysis to examine the linear relationships between psychological outcomes and decision-making self-efficacy. This approach provides foundational insight prior to more complex multivariate modeling that will be implemented in future studies.

**Table 3:** Relationship between DASS 21 and self-efficacy.

	Mean	*SD*	Pearson Correlation	*P* Value
Self-efficacy	41.29	16.15	0.008	0.943
Depression	18.31	11.11		
Self-efficacy	41.29	16.15	–0.015	0.891
Anxiety	17.04	11.75		
Self-efficacy	41.29	16.15	0.066	0.534
Stress	19.47	11.60		
Self-efficacy	41.29	16.15	0.021	0.844
Total DASS-21	54.82	32.45		

## Discussion

The current study aimed to investigate the psychological outcomes (depression, anxiety, and stress) among family members of critically ill patients in Saudi Arabia, as well as to explore the relationship between these outcomes and decision-making self-efficacy. Although the hypothesis anticipated a significant inverse relationship between psychological distress and decision-making self-efficacy, these unexpected results suggest that other variables, such as perhaps resilience, coping strategies, or supportive family and community networks, may mediate the link between psychological distress and decision-making self-efficacy.

Regarding the prevalence of psychological outcomes, the results showed a high prevalence of depression, anxiety, and stress among family members. These findings align with previous research highlighting the emotional distress experienced by families in similar situations [9,10,18]. One possible explanation is that families play a central role in providing support and care for their loved ones. The emotional and psychological impact on family members when faced with critical illness within the family can be particularly pronounced in Saudi Arabian culture. According to Abdul Halain et al. (2022), high incidences of anxiety and depression were also reported for the family members of ICU patients (18), which corroborate the present study’s findings. To further enhance the clinical interpretation of these results, Xing et al. (2024) noted that DAs increase family members’ knowledge and reduce decisional conflict, with inconclusive effects on anxiety and depression (19). Hence, the above studies drew a comparison with the findings of the present studies, emphasizing the importance of family-centered interventions, communication skills training, and structured decision-making aid to lessen psychological distress.

Saudi Arabian culture places a strong emphasis on collectivism, such that individuals prioritize the needs and well-being of the family unit over individual concerns [19]. This cultural value may contribute to the high levels of psychological distress experienced by family members, because they may feel a strong sense of responsibility and obligation toward their ill loved ones. Additionally, cultural norms and expectations regarding gender roles and familial duties may further amplify the emotional burden on family members.

An examination of the relationship between psychological outcomes and decision-making self-efficacy found no significant correlation. This finding contradicts previous evidence [20,21], which suggested a link between psychological outcomes and decision-making self-efficacy. One potential explanation for this inconsistency could be differences in research design, because previous studies used qualitative methods such as focused group interviews, whereas the current study employed quantitative measures. Moreover, these findings highlight the complexity of the relationship between psychological well-being and decision-making self-efficacy. They suggest that although individuals may experience symptoms of depression, anxiety, or stress, their experience does not necessarily undermine their belief in their own decision-making abilities. In addition, this study did not explore other important factors such as personal resilience, coping strategies, support systems, and personal beliefs that could have a big impact on a person’s decision-making confidence.

## Limitations and future research

There are several limitations to this exploratory study. First, the study design was cross-sectional and does not provide information regarding the direction or causality of associations. Although statistically significant correlations can demonstrate that relationships exist between two variables, only longitudinal designs and/or advanced models (i.e., regression or structural equation modeling) can provide the evidence needed to support causal pathways.

Second, the participants constituted a relatively small sample size and one cultural context and, therefore, generalizability is limited. Future studies should use larger and more diverse recruitment strategies (such as the idea of the minimum standard of at least recruiting 200–300 participants that is suggested by similar ICU-family research) in order to provide more statistical power and cultural representativeness.

Third, all measures relied on self-report and could be impacted by social desirability and/or emotional fatigue. Ideally, investigator triangulation using clinician-rated measures or qualitative interviews could provide some additional validity.

Fourth, while demographic variables were collected, they were not explored as moderators. Future research should look at whether gender, education, or consciousness levels of the patient influence the relationship between psychological distress and decision-making self-efficacy.

Lastly, as data collection occurred during the COVID-19 pandemic, participants may have levels of distress that are higher than normal and run the risk of inflating their scores in psychological measures. Considering comparisons post-pandemic would provide more generalizable benchmarks.

## Conclusions

This exploratory study examined psychological outcomes and decision-making self-efficacy among family members of critically ill patients in Saudi Arabia. The study contributes original data in a context that remains underrepresented in global critical care literature. While no significant correlations were found, the findings underscore the high prevalence of psychological distress and the need for culturally adapted support interventions.

Future research should investigate potential mediating factors such as resilience, coping styles, and social support. Furthermore, multivariate and longitudinal designs could better capture the complex interplay between emotional distress and decision-making in high-pressure medical environments.
