# Correction: An exploratory study of psychological and decision-making outcomes among family members of critically ill patients

**DOI:** 10.1371/journal.pone.0339802

**Published:** 2025-12-29

**Authors:** Marym M. Alaamri, Shoroug Shaker Darweesh, Sarah Wasl Algabasani, Sarah Mohammad Alsubiei, Nora Jaber Alsolami, Hanan Mohsen Alhaddad, Afnan Tunsi, Aisha Alhofaian

There is an error in affiliation of the authors. The correct affiliation is: Department Medical Surgical Nursing, Faculty of Nursing, King Abdulaziz University, Jeddah, Saudi Arabia.
